# A Mobile Health App (Roadmap 2.0) for Patients Undergoing Hematopoietic Stem Cell Transplant: Qualitative Study on Family Caregivers' Perspectives and Design Considerations

**DOI:** 10.2196/15775

**Published:** 2019-10-24

**Authors:** Dima Chaar, Ji Youn Shin, Amanda Mazzoli, Rebecca Vue, Jacob Kedroske, Grant Chappell, David A Hanauer, Debra Barton, Afton L Hassett, Sung Won Choi

**Affiliations:** 1 University of Michigan School of Public Health Department of Epidemiology Ann Arbor, MI United States; 2 Michigan State University College of Communication Arts and Sciences Department of Media and Information East Lansing, MI United States; 3 University of Michigan Medical School Blood and Marrow Transplantation Program Ann Arbor, MI United States; 4 University of Michigan Medical School Michigan Institute for Clinical and Health Research Ann Arbor, MI United States; 5 University of Michigan School of Nursing Ann Arbor, MI United States; 6 University of Michigan Medical School Department of Anesthesiology Ann Arbor, MI United States

**Keywords:** caregivers, allogeneic hematopoietic stem cell transplant, home interviews, user experience, mobile health apps

## Abstract

**Background:**

Hematopoietic stem cell transplantation (HCT), also referred to as blood and marrow transplantation (BMT), is a high-risk, but potentially curative therapy for a number of cancer and noncancer conditions. BMT Roadmap (Roadmap 1.0) is a mobile health app that was developed as a family caregiver–facing tool to provide informational needs about the health status of patients undergoing inpatient HCT.

**Objective:**

This study explored the views and perceptions of family caregivers of patients undergoing HCT and their input regarding further technology development and expansion of BMT Roadmap into the outpatient setting (referred to as Roadmap 2.0).

**Methods:**

Semistructured qualitative interviews were conducted among 24 family caregivers. Questions were developed from existing literature coupled with prior in-depth observations and interviews in hospital-based settings to explore the study objectives. Participants were recruited during routine outpatient clinic appointments of HCT patients, and all interviews were conducted in the participants’ homes, the setting in which Roadmap 2.0 is intended for use. A thematic analysis was performed using a consistent set of codes derived from our prior research. New emerging codes were also included, and the coding structure was refined with iterative cycles of coding and data collection.

**Results:**

Four major themes emerged through our qualitative analysis: (1) stress related to balancing caregiving duties; (2) learning and adapting to new routines (resilience); (3) balancing one’s own needs with the patient’s needs (insight); and (4) benefits of caregiving. When caregivers were further probed about their views on engagement with positive activity interventions (ie, pleasant activities that promote positive emotions and well-being such as expressing gratitude or engaging in activities that promote positive thoughts, emotions, and behaviors), they preferred a “menu” of positive activities to help support caregiver health and well-being.

**Conclusions:**

This study involved family caregivers as participants in the development of new components for Roadmap 2.0. Our research provided a further understanding of the many priorities that hematopoietic stem cell transplant family caregivers face while maintaining balance in their lives. Their schedules can often be unpredictable, even more so once the patient is discharged from the hospital. Our findings suggest that expanding Roadmap 2.0 into the outpatient setting may provide critical caregiver support and that HCT caregivers are interested in and willing to engage in positive activities that may enhance well-being and attenuate the stress associated with caregiving.

**International Registered Report Identifier (IRRID):**

RR2-10.2196/resprot.4918

## Introduction

### Family Caregivers in the Clinical Context of Hematopoietic Stem Cell Transplantation

Hematopoietic stem cell transplantation, also commonly referred to as blood and marrow transplantation (BMT), is a high-risk, but potentially curative therapy for a number of cancer and noncancer conditions [1]. Given the high-risk associated with this treatment modality, patients require a dedicated family (or friend) caregiver who is available full time (24/7) for at least the first 100 days after the transplant [2]. The caregiver must receive medical training on catheter line care, intravenous and oral medication management, identification of signs and symptoms of infection or other posttransplant complications, and management of care and activities of daily living. Significant levels of anxiety and distress are experienced by caregivers during the peritransplant period (ie, pretransplant and immediately following discharge from the hospital for the hematopoietic stem cell transplant procedure) [3]. The stress and demands of caregiving for HCT patients may be further heightened if patients and caregivers are required to relocate to be closer to the hospital. This burden is associated with a significant decline in emotional, physical, and social functioning of the caregiver [4-7]. The reciprocal relationship between patient and caregiver emotional distress strongly supports the need to promote caregiver health and well-being [8].

### Need for Novel Caregiver-Based Interventions

Receiving education on patient illness has been identified as an approach for reducing stress and anxiety [9,10]. Complex medical tasks are initially performed by a professional nurse in the hospital setting, with responsibility for these tasks transferred to the caregiver to perform at home. Accordingly, the caregiver needs education about the diagnosis and prognosis of the patient (knowledge), training in medical care (skills), and information about available resources to achieve patient and caregiver-centered care [11], which may protect the caregiver from harm by increasing their health and well-being [12]. Thus, novel interventions that provide caregiver support are urgently needed [13].

### Mobile Health Technology: BMT Roadmap

BMT Roadmap (Roadmap 1.0) was developed as a caregiver-facing mobile health app to provide information, education, and skills building to meet informational needs during the inpatient transplant process (Multimedia Appendix 1) [14-19]. Screenshots of the app are provided in Multimedia Appendix 2. Patients, caregivers, and health care providers actively participated in iterative cycles of user-centered design, development, and testing, which has been shown to be crucial for the correct use and adoption [20]. To date, >100 HCT caregivers and patients have consented and enrolled in institutional review board–approved studies to assess the feasibility of implementing Roadmap 1.0 during the inpatient setting [21]. Patient-reported outcomes were collected at baseline, discharge, and day 100 in a pre- and poststudy design.

Roadmap 1.0 was designed as a multicomponent app that included the following modules: an overview of the criteria needed for discharge along with training for self-management, which was required for discharge (eg, videos of central line care); real-time laboratory results graphed so that visual trends could be tracked longitudinally across time; personalized medication lists with common indications, dosing and schedule, and side effects; health care provider “Facebook” photos; details about clinical trials that patients were enrolled in; and educational materials defining commonly used medical terms and concepts that the medical team discusses with families. Caregivers were instructed to use it freely throughout their hospital stay.

Roadmap 1.0 was rated as highly useful and easy to use [22,23]; caregiver anxiety [24] decreased; caregiver health-related quality of life [25] improved; and caregiver activation [26] increased at discharge compared with baseline [22]. The most used modules were laboratory studies and medications, which were rated highly useful and easy to understand and visualize. In addition, weekly semistructured qualitative interviews were conducted on topics including acceptance of and barriers to Roadmap 1.0, informational needs, and psychosocial burdens. Major themes emerging from content analyses using memos and coding included the following: Roadmap 1.0 had high usefulness, ease of use, and likeability; its laboratory module was the most viewed; and participants expressed a desire for additional components along with expansion of Roadmap 1.0 into the outpatient setting. The interviews also highlighted the need for supporting caregiver health and well-being, but also showed the positive aspects of caregiving, such as new or closer relationships with loved ones. Collectively, these findings support the rationale to extend Roadmap to the outpatient setting. Furthermore, caregivers expressed modules that include caregiver-specific resources as well as positive activities that they could perform throughout the transplant trajectory, since it does not end after discharge from the hospital.

This study aimed to explore the views and perceptions of HCT caregivers for expanding Roadmap 1.0 into the home environment, outside of the hospital, where the app would be most used (the expanded version of the app will be referred to as Roadmap 2.0 henceforth). We were also interested in examining the life of a caregiver in her/his home environment to inform Roadmap 2.0.

## Methods

### Study Site

The field sites in this study included participant’s homes, defined as permanent residence, rental apartment, and local extended-stay accommodations (eg, motels, hotels, and suites). The homes were all within approximately 90 minutes of travel distance from the University of Michigan, a large, tertiary academic medical center, as mandated by the transplant program.

### Study Design, Recruitment, and Consent

Semistructured interviews were conducted among 24 family caregivers. Eligibility for study participation included the following: primary family caregiver who had already experienced the transplant procedure with their loved one (patient) and was in the posttransplant phase of care; age≥18 years; comfortable with reading and speaking English; willing to participate in a face-to-face interview in a home setting; and able to provide informed consent. Participants were recruited through referrals from the hematopoietic stem cell transplant clinical team and included caregivers of transplant patients visiting the hospital for routine outpatient clinic appointments. The hematopoietic stem cell transplant clinical team notified the research team if the caregiver agreed to learn more about the study. All caregivers approached for the study agreed to participate. One caregiver was approached by the study coordinator, but was deemed ineligible because English was not her primary language and she was not comfortable with reading and speaking English. Our primary dataset included semistructured interviews with the primary caregivers of hematopoietic stem cell transplant patients (ie, provided ≥50% of caregiving responsibilities). Saturation is defined in qualitative research as a criterion for discontinuing data collection and/or analysis [27]. Recruitment ended once it was determined that no additional data were being found, through which the investigators could develop new thematic categories.

Ethical approval for this study was obtained by the Institutional Review Board. All participants signed an informed consent prior to the research investigators traveling to their residence. Once the informed consent document was signed, the research team worked with the participant to arrange for a date and time that worked well for both. All interviews were conducted in the participants’ homes. A minimum of two research assistants traveled to the homes. The home semistructured interview questions were informed by existing literature as well as prior observations and interviews conducted in hospital-based settings (inpatient and outpatient transplant units). The questions were subsequently optimized by the study team. Approximately 250 hours of hospital-based observations and interviews were conducted by a minimum of two research assistants (SW Choi, unpublished data, 2019). Observations were recorded as field notes, and interviews were audio-recorded, with permission, and subsequently professionally transcribed (Babbletype LLC, Philadelphia, Pennsylvania). The home interviews explored several domains including demographic information, general caregiver duties after transplant, posttransplant life experiences, use of technology and social media platforms to support caregiver self-management and provide informational resources, and positive activity interventions to promote caregiver health and well-being (Multimedia Appendix 3).

### Positive Activity Interventions

Based on prior in-depth observations and interviews conducted in the hospital of Roadmap 1.0, we found that caregivers were interested in and willing to engage in positive activity interventions. It was acknowledged that caregiving can be stressful and personal well-being needed to be attended to in order to best support the patient. Thus, in this study, positive activity interventions were defined to caregivers as simple pleasant activities that were intentional and could be developed into routine practices such as expressing gratitude or scheduling activities that promoted positive thoughts, emotions, or behaviors [28-30]. Artifacts, such as photographs, that reflected the caregiver home environment were also collected during home interviews in efforts to inform Roadmap 2.0.

### Participant Demographics

The median age of the study participants was 57 years (range, 31-71 years). The majority were married or in a domestic partnership (23/24 [96%]), white (23/24 [96%]), and female (20/24 [83%]). In addition, 20/24 (83%) received at least a 2-year college degree and 12 participants (50%) received at least a 4-year college degree. The interviews took place in permanent residences (11/24 [46%]), rental apartments (10/24 [42%]), or local accommodations (3/24 [12%]; Multimedia Appendix 4).

### Thematic Analysis

The semistructured interviews were conducted one-to-one by research investigators who were trained in qualitative methods with experience working in the hematopoietic stem cell transplant population. The interviews lasted about 60-80 minutes. The interview script was developed by DC and JS, and minor iterations were made to adjust for clarity of the questions after piloting the home interview script with the rest of the research team (SC, DH, DC, JK, RV, and GC; Multimedia Appendix 5). The sentence structures were refined to make the question as well as potential probing questions clear. All qualitative semistructured interviews were audio-recorded, deidentified, and entered into NVivo Pro 11 (QSR International, Melbourne, Australia). Data were analyzed using a thematic approach, in that our data collection and analyses mutually informed one another. During the first stage of data analysis, initial interviews were professionally transcribed verbatim, followed by qualitative content analysis [31] of the data using an open coding method. The transcripts were coded according to each concept (ie, line by line or by paragraph) using NVivo Pro 11 software. The research team (SC, DC, JS, JK, RV, and GC) then collectively generated new codes as significant concepts, and patterns of data (themes) were identified, discussed, and revised. All the members who participated in the thematic analysis followed the phases of thematic analysis: (1) familiarized her/himself with the data; (2) generated initial codes; (3) searched for themes; (4) reviewed themes; (5) defined and named the themes; and (6) produced the report [32].

The second stage of data analysis, using new interview as well as artifact data that emerged, resulted in consistent themes and confirmed our findings. A minimum of three team members took an active role in identifying patterns/themes based on how the data were classified by codes. Common structures and themes that arose repeatedly through content analysis were identified and organized hierarchically by patterns or structures in the code [33], which were subsequently further grouped together in overarching themes. The final interpretation of codes and themes were completed by the first and second authors and two senior researchers (DC, JS, DH, SC). The semistructured interview script and code book are provided in Multimedia Appendices 5 and 6, respectively. Texts or artifacts were gathered from participant homes and analyzed with the qualitative data [34] to further inform the themes that were emerging.

## Results

### Overview

Four overarching themes emerged through 37 codes that developed out of our thematic qualitative analyses: (1) stress related to balancing caregiving duties, (2) learning and adapting to new routines (resilience), (3) balancing one’s own needs with the patient’s needs (insight), and (4) benefits of caregiving.

### Theme 1: Stress Related to Balancing Caregiving Duties

Once patients were discharged home, caregivers reported heightened anxiety of the new duties and tasks as well as being in the small living conditions of temporary housing (eg, rental apartment or hotel room; Multimedia Appendix 7). After a prolonged hospital course, caregivers described further time away from their (permanent resident) homes and support system as being isolating. Caregivers were suddenly thrust into multitasking and balancing caregiving duties such as cleaning, providing transportation, planning meals, scheduling appointments, administering/organizing medications, and performing medical tasks (eg, central line care and infusions). Representative quotes are shown in Multimedia Appendix 8.

### Theme 2: Learning and Adapting to New Routines (Resilience)

Caregivers reported that while they initially felt overwhelmed when they first came home after a prolonged transplant course in the hospital, they adjusted to the new routine of home care. Caregivers reported their ability to perform complex and demanding tasks that gradually improved over time through repeating the skill (eg, medication administration and dressing changes). A representative participant quote is shown in Multimedia Appendix 8. Caregivers developed their own processes for organizing medications according to time of administration (eg, morning, afternoon, and evening) (Multimedia Appendix 7); caregivers scheduled reminders in calendars (on paper) or used mobile devices to set alarms; caregivers saved business cards of various health care providers and taped them to their walls (Multimedia Appendix 7); caregivers also posted discharge contact information as well as local area dining and shopping options (Multimedia Appendix 7).

Despite the burden of these new duties and routines, some caregivers voiced optimism and strength. Having beautiful flowers or Get Well cards in the room provided encouragement and support (Multimedia Appendix 7). For example, despite the medically complex and emotionally difficult treatment process endured by the patient, a caregiver described it as “temporary” and the cancer diagnosis being a “bump in the road.” Caregivers displayed resiliency in the midst of the transplant journey as they learned to adapt to the requirements of caregiving and shared new experiences with family or friends (Multimedia Appendix 8).

### Theme 3: Balancing One’s Own Needs With the Patient’s Needs (Insight)

Caregivers gained insight by recognizing the need to care for their own health while also serving their loved ones. In addition to developing new routines on behalf of the patient, many were also adjusting to living in new, temporary accommodations in order to be closer to the hospital, as mandated by the transplant team. More than half of the study participants (52%) were not living in their permanent residence. As such, caregivers developed new strategies over the transplant trajectory that focused on themselves as well as the patients. Many of these strategies included self-care or management of their own stress that enabled them to be “better” caregivers. Some examples that were provided included taking a quiet moment for themselves (ie, quiet time or moments of solitude), focusing on sleep, maintaining pre-existing relationships or friendships, and engaging in sports or physical activity (eg, golf and yoga). One participant noted that simply getting out of the house was useful (Multimedia Appendix 8).

### Theme 4: Benefits of Caregiving

Caregivers made considerable adjustments in their daily lives. Some caregivers took time off work and had not yet returned to work at the time of interviews. Nonetheless, caregivers found meaning in performing their medically related duties on behalf of the patient or simply from spending time with the patient in “isolated” environments (ie, minimizing visitors or going to the grocery store during early morning or later evening hours to mitigate infectious contacts). Caregivers noticed that they found benefits in caregiving, such as deeper compassion and empathy toward the patient; they identified the positive aspects of caregiving. For example, while one caregiver shared that she now had to rely on other family members, this allowed deeper relationships to form (Multimedia Appendix 8). Another caregiver expressed how journaling provided an opportunity to reflect on the transplant journey (Multimedia Appendix 7).

### Positive Activity Interventions

Caregivers were asked to rank a particular positive activity on a scale of 1-10, with the higher number indicating that the activity could be very beneficial (Multimedia Appendix 3). Pleasant Activity Scheduling, where participants would set aside time each day for a positive activity (eg, having ice cream with a friend or taking a walk in the park) was ranked the highest. This activity was followed by Gratitude journaling (ie, writing down things for which they were grateful) and Savoring the Moment (ie, being mindful of and savoring a pleasant experience, such as drinking coffee or spending time with a friend, and using all of the senses to solidify the memory; Table 1). When reflecting on the possible benefits of the Savoring the Moment positive activity, one of the caregivers shared how he was already incorporating this activity to his early mornings and found it helpful (Multimedia Appendix 8).

**Table 1:** Engagement with positive activity interventions.

Positivity activity	Median Likert rating (range)^a^
Pleasant Activity Scheduling	9.0 (5.0-10.0)
Gratitude Journaling	10.0 (3.0-10.0)
Savoring	9.0 (4.0-10.0)
Random Acts of Kindness	8.0 (2.0-10.0)
Positive Piggy Bank	6.0 (1.0-10.0)
Signature Strengths	6.5 (1.0-9.0)

^a^Scale: 1-10; 1=not likely to engage in the activity; 10=very likely to engage in the activity.

## Discussion

### Main Findings

In this study, we explored the views and perceptions of HCT caregivers to expand Roadmap 2.0 into the home environment. Through our qualitative findings, four major themes emerged: (1) stress related to balancing caregiving duties; (2) learning and adapting to new routines (resilience); (3) balancing one’s own needs with the patient’s needs (insight); and (4) benefits of caregiving. These findings support the importance of linking caregivers to resources throughout the transplant trajectory, beyond the hospital course, because in many instances, caregivers are not even aware of the support services available. The artifacts collected (eg, photographs) and semistructured interviews in caregivers’ home environment provided content and design considerations for the outpatient version of Roadmap 2.0; suggested topics or categories are provided in Multimedia Appendix 3.

Caregivers in this study gained new insights and recognized the importance of maintaining their own health and well-being, while also caring for the patient. Accordingly, they indicated the willingness to engage in positive activity exercises. The research team asked the caregivers to rank each activity on a Likert scale. Caregivers reflected on their own subjective values and experiences in determining the ranking. In general, they preferred having a “menu” or choice of a variety of positive activity exercises. The majority favored Pleasant Activity Scheduling, where caregivers would set aside time each day for a positive activity. This was followed by Gratitude journaling and Savoring the Moment, a mindfulness-based exercise. Interestingly, these activities correlated with the examples caregivers provided when discussing strategies employed while caring for the patient (ie, setting aside time for themselves).

### Positive Aspects of Caregiving

Increasing amounts of data suggest that simple strategies aimed at enhancing positive thoughts, emotions, and behaviors are effective and highly scalable [28-30]. Positive activity interventions such as daily positive reflection, gratitude journals, and conducting acts of kindness have been used in other medical populations including those with cardiovascular disease, cancer, diabetes, and chronic pain [35-38], but not in caregivers of HCT patients. Our preliminary data suggest that caregivers desire the opportunity to try these activities to reduce stress and improve their health-related quality of life. Research has shown that much focus has been placed on the wide range of negative implications associated with caregiving [4], such as increases in depression, anxiety, and other health-related concerns [39]. Despite this, a majority of caregivers have recognized the benefits of caregiving [40,41].

Studies have shown that positive psychology interventions enhance subjective and psychological well-being and help reduce depressive symptoms in a cost-effective and targeted manner, particularly when combined with other evidence-based interventions [29]. Further, research supports implementing a selection of positive activity exercises as opposed to introducing only one activity (ie, Gratitude journal, Random Acts of Kindness, and Signature Strengths vs Gratitude journal only). Our caregiver interviews herein highlight the importance that “one size does not fit all,” despite the relatively homogenous participant population. It is possible that caregivers who are able to choose their positive activity exercise may want to engage in them longer and frequently. Nonetheless, research indicates that the ideal dosage and timing as well as combination of activities remain unclear [42].

### Home Environment as a Novel Study Site to Explore Caregivers’ Views

HCT caregiving is intense and complex with unexpected fluctuations throughout the transplant trajectory. This paper has specifically focused on descriptive qualitative approaches conducted in the home setting of HCT caregivers. We focused on the home environment, which was informed from prior observational and qualitative work in the hospital. Since the early prototype of Roadmap 1.0 [43,44], we have continued to incorporate key stakeholders (eg, patients, caregivers, physicians, nurses, psychologists, and social workers) in its design and development [43,44]. Importantly, the iterative process has included a multidisciplinary team. In the home environment, it was apparent that HCT caregivers face competing demands related to caregiving while maintaining other life duties (ie, self-care, home care, work, and other children, spouse, or parent care). More strikingly, even after hospital discharge, more than half our HCT caregivers were living in rental apartments or local accommodations. The unfamiliar and isolated environment critically speaks to novel and innovative solutions that are needed to support them beyond the hospital setting. We need to design, develop, and test interventions that support caregiver health and well-being in the midst of multitasking and balancing duties while further strengthening their resiliency. Mobile health technology has the potential to deliver scalable solutions [45-48]. Although HCT caregivers sought out new or different relationships with family members or friends, providing (reputable or health care provider-approved) caregiver-specific resources may provide benefit.

### Limitations and Future Directions

Our work supports caregivers as key stakeholders in the development of mobile health technology for the provision of patient care. Although we recognize the limitations of this study, such as single-center design, and relatively homogeneous participant population (eg, white, female, educated) with bias toward willingness to participate, the strengths of the study include rigorous data collection and analyses and novel study site (home environment). Based on our findings, we have constructed a Caregiver Health Survey to quantify the views and perspectives of HCT caregivers nationally, which will capture a larger, more diverse population. We hope that our collective qualitative and quantitative findings will inform the design and development of a positive psychology intervention to provide caregiver support in the outpatient setting. The multicomponent intervention will include a “menu” of positive activities that will be tested in a randomized controlled trial design.

## Appendix

Multimedia Appendix 1 BMT Roadmap components.

Multimedia Appendix 2 Screenshots of BMT Roadmap laboratory studies and medications modules.

Multimedia Appendix 3 Caregiver-specific resources and positive activities components.

Multimedia Appendix 4 Participant demographics.

Multimedia Appendix 5 Home interview script.

Multimedia Appendix 6 Codebook.

Multimedia Appendix 7 Artifacts collected in the home environment.

Multimedia Appendix 8 Participant quotes.

## Abbreviations

BMT: blood and marrow transplantation
HCT: hematopoietic stem cell transplant
