# Supplementary material for: A Mobile Health App (Roadmap 2.0) for Patients Undergoing Hematopoietic Stem Cell Transplant: Qualitative Study on Family Caregivers' Perspectives and Design Considerations
Source: JMIR Mhealth Uhealth. 2019 Oct 24;7(10):e15775. doi: 10.2196/15775 (PMC6913725; doi:10.2196/15775)
Supplement: Multimedia Appendix 1 [file mhealth_v7i10e15775_app1.pdf]

Multimedia Appendix 1

BMT Roadmap Components	
**Patient-Specific Informational Resources**	
**Disease Characteristics**	Infectious disease markers, blood type, donor type, donor characteristics, chemotherapy regimen
**Laboratory Studies**	Results of laboratory studies updated in real-time
**Medications**	Medication list grouped according to indication (i.e., antibiotic, anti-emetic), dosing and schedule
**Clinical Trials**	Easy-to-read description of clinical trials and copies of consent
**Provider Directory**	Photos of healthcare providers in yearbook style
**Caregiver-Specific Positive Activities**	
**Glossary**	Commonly used words/terms and definitions used the transplant journey
**Phases of Care**	Roadmap metaphor with detailed description of each phase of transplant
**Symptoms/Side Effects**	Description of commonly reported medication side effects
**Patient-Specific Skills Building**	
**Videos**	Video modules of central line care, dressing changes, cleaning, bathing
**Discharge Checklist**	Interactive list of 9-item discharge criteria to assess “readiness”
