# Supplementary figures and images for: A Mobile Health App (Roadmap 2.0) for Patients Undergoing Hematopoietic Stem Cell Transplant: Qualitative Study on Family Caregivers' Perspectives and Design Considerations
Source: JMIR Mhealth Uhealth. 2019 Oct 24;7(10):e15775. doi: 10.2196/15775 (PMC6913725; doi:10.2196/15775)

Multimedia Appendix 2

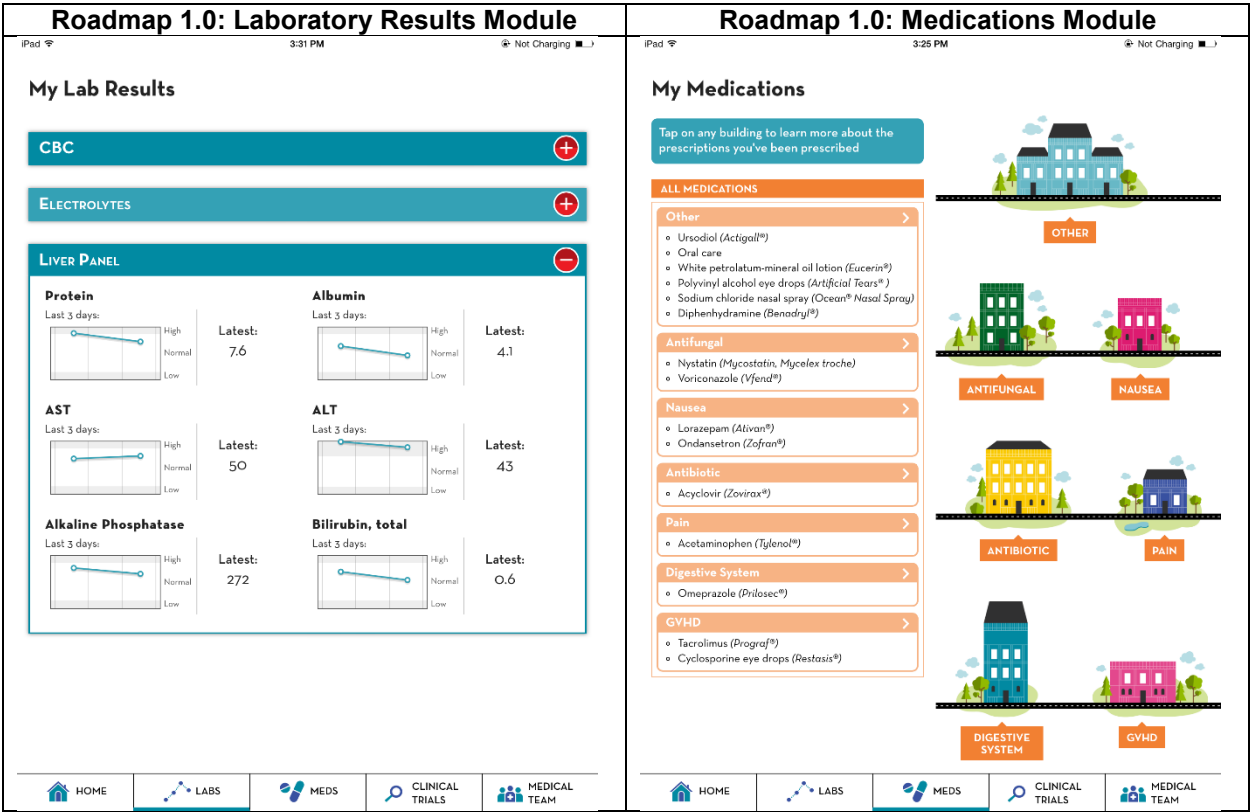

Supplement: Multimedia Appendix 2 [file mhealth_v7i10e15775_app2.pdf]
