# Supplementary material for: A Mobile Health App (Roadmap 2.0) for Patients Undergoing Hematopoietic Stem Cell Transplant: Qualitative Study on Family Caregivers' Perspectives and Design Considerations
Source: JMIR Mhealth Uhealth. 2019 Oct 24;7(10):e15775. doi: 10.2196/15775 (PMC6913725; doi:10.2196/15775)
Supplement: Multimedia Appendix 3 [file mhealth_v7i10e15775_app3.pdf]

Multimedia Appendix 3

Caregiver-Specific Resources and Positive Activities Components	
**Caregiver-Specific Resources**	
**Peer-to-Peer Contact**	BMT Roadmap to connect families; Resource links to BMT InfoNet or nbmtLink to connect families
**Coping Techniques**	Instructions on self-care, meditation and relaxation techniques (positive coping skills: write, draw, paint)
**Intrinsic Motivation**	Instructions to help caregivers identify goals (values framing)
**Physical Activity Tips**	Instructions of exercises of varying intensity; walking/jogging course
**Healthy Sleeping Tips**	Instructions on healthy sleeping principles (sleep journal)
**Caregiver-Specific Positive Activities**	
**Positive Piggy Bank**	Every evening, the family caregiver will think about the things that made her/him happy; write down one of these moments on a piece of paper, fold up the piece of paper and drop it in the piggy bank. At the end of 30 days, the caregiver will "close the account" and open the piggy bank and read and savor all of the deposited memories.
**Pleasant Activity Scheduling**	Set aside a small block of time each day for a positive activity (e.g., watching a favorite show, bubble bath, having ice cream with a friend). This is an "appointment" just as serious as any other appointment.
**Gratitude Journal**	Every day for 30 days the caregiver will write down 3 things for which the caregiver is grateful – 3 new things/day.
**Random Acts of Kindness**	One day of the week, do 5 acts of kindness – including something kind for oneself. Self-care is important (e.g., bubble bath, massage, walk in park, popsicle).
**Savoring**	The caregiver will be asked to spend a few minutes each day savoring two everyday experiences (e.g., coffee, sunshine, call from friend). The caregiver is to be mindful while savoring the experience and use all of the senses to solidify the memory.
**Signature Strengths**	The caregiver will identify the top 7 character strengths using a brief questionnaire (e.g., kindness, creativity, perseverance, bravery, intelligence). The caregiver will use one of these strengths in a new way every day over a week.
