# Supplementary material for: A Mobile Health App (Roadmap 2.0) for Patients Undergoing Hematopoietic Stem Cell Transplant: Qualitative Study on Family Caregivers' Perspectives and Design Considerations
Source: JMIR Mhealth Uhealth. 2019 Oct 24;7(10):e15775. doi: 10.2196/15775 (PMC6913725; doi:10.2196/15775)
Supplement: Multimedia Appendix 4 [file mhealth_v7i10e15775_app4.pdf]

## Multimedia Appendix 4

Participant demographics

| ID | Age (of Patient) at HCT | Relationship | Gender of Caregiver | Age | Race | Spanish, Hispanic or Latino origin | Marital Status | Highest level of schooling | Current employment status | Annual house hold income | Staying At Hotel vs Apartment vs Home |
|---|---|---|---|---|---|---|---|---|---|---|---|
| CG01 | 20 | Mother | Female | 57 | White | No | Married or in domestic partnership | Some college or 2-year degree | Employed part time (up to 39hrs/week) | $60,001-100,000 | Apartment |
| CG02 | 24 | Mother | Female | 55 | White | No | Married or in domestic partnership | High school graduate or GED | Unemployed and currently looking for work | $60,001-100,000 | Home |
| CG04 | 63 | Family Friend | Female | 58 | White | No | Married or in domestic partnership | More than 4-year college degree | Retired | $0 - $30,000 | Apartment |
| CG05 | 62 | Wife | Female | 57 | White | No | Married or in domestic partnership | Some college or 2-year degree | Employed full-time (40 or more hours per week) | $60,001-100,000 | Apartment |
| CG07 | 69 | Wife | Female | 68 | White | No | Married or in domestic partnership | High school graduate or GED | Retired | $30,001-$60,000 | Home |
| CG08 | 53 | Husband | Male | 53 | White | No | Married or in domestic partnership | More than 4-year college degree | Employed full-time (40 or more hours per week) | Greater than $200,000 | Home |
| CG09 | 11 | Mother | Female | 53 | White | No | Married or in domestic partnership | 4-year college graduate | Unable to work | $0 - $30,000 | Hotel |
| CG10 | 31 | Mother | Female | 62 | Black or African American | No | Married or in domestic partnership | Some college or 2-year degree | Employed full-time (40 or more hours per week) | $30,001-$60,000 | Apartment |
| CG11 | 65 | Husband | Male | 60 | White | No | Married or in domestic partnership | 4-year college graduate | Retired | $60,001-$100,000 | Home |
| CG12 | 66 | Wife | Female | 56 | White | No | Married or in domestic partnership | More than 4-year college degree | Unemployed and not currently looking for work | $30,001-$60,000 | Apartment |

| | | | | | | | | | | | |
|---|---|---|---|---|---|---|---|---|---|---|---|
| CG13 | 43 | Family Friend | Female | 44 | White | no | Married or in domestic partnership | Some college or 2-year degree | Retired | $60,0001 - $100,000 | Apartment |
| CG14 | 64 | Sister | Female | 60 | White | no | Married or in domestic partnership | More than 4-year college degree | Retired | $60,001-$100,000 | Hotel |
| CG15 | 65 | Wife | Female | 63 | White | No | Married or in domestic partnership | Some college or 2-year degree | Retired | $30,001 - $60,000 | Apartment |
| CG16 | 53 | Husband | Male | 53 | White | No | Married or in domestic partnership | 4-year college graduate | Employed full-time (40 or more hours per week) | Prefer not to answer | Home |
| CG17 | 66 | Family Friend | Female | 62 | White | No | Divorced | Some college or 2-year degree | Self-Employed/ Unable to Work | $0-$30,000 | Home |
| CG18 | 59 | Daughter | Female | 31 | White | No | Married or in domestic partnership | More than 4-year college degree | Employed part time (up to 39hrs/week) | $100,001 - $200,000 | Home |
| CG19 | 37 | Wife | Female | 35 | White | No | Married or in domestic partnership | More than 4-year college degree | Employed full-time (40 or more hours per week) | $100,001 - $200,000 | Home |
| CG20 | 44 | Mother | Female | 70 | White | No | Married or in domestic partnership | High school graduate or GED | homemaker | $30,001-$60,000 | Apartment |
| CG21 | 64 | Wife | Female | 59 | White | No | Married or in domestic partnership | High school graduate or GED | Unemployed and not currently looking for work | $30,001-$60,000 | Apartment |
| CG22 | 43 | Wife | Female | 39 | White | No | Married or in domestic partnership | More than 4-year college degree | Employed full-time (40 or more hours per week) | $60,001-$100,000 | Home |
| CG23 | 67 | Family Friend | Female | 56 | White | No | Married or in domestic partnership | Some college or 2-year degree | Employed part time (up to 39hrs/week) | $100,001 - $200,000 | Apartment |
| CG24 | 25 | Father | Male | 71 | White | No | Married or in domestic partnership | More than 4-year college degree | Retired | $100.001 - $200,000 | Home |

| | | | | | | | | | | | |
|---|---|---|---|---|---|---|---|---|---|---|---|
| CG25 | 58 | Wife | Female | 58 | White | No | Married or in domestic partnership | Some college or 2-year degree | Employed part time (up to 39hrs/week) | $60,001-$100,000 | Hotel |
| CG26 | 57 | Wife | Female | 57 | White | No | Married or in domestic partnership | 4-year college graduate | Employed full-time (40 or more hours per week) | $100,001 - $200,000 | Home |
