# Supplementary material for: A Mobile Health App (Roadmap 2.0) for Patients Undergoing Hematopoietic Stem Cell Transplant: Qualitative Study on Family Caregivers' Perspectives and Design Considerations
Source: JMIR Mhealth Uhealth. 2019 Oct 24;7(10):e15775. doi: 10.2196/15775 (PMC6913725; doi:10.2196/15775)
Supplement: Multimedia Appendix 5 [file mhealth_v7i10e15775_app5.pdf]

Multimedia Appendix 5

Home Interview Script

Demographic Information

1. Can you please tell me about yourself? (Specifically: What's your occupation? Do you have any past experience as a caregiver?)

General information after transplant

2. Are you his/her primary caregiver? Do you have a secondary support system in place? Would you be interested/ comfortable in an at home nurse or student caregiver taking over once a week or as needed?
3. Tell me about your journey through this process after your loved one was diagnosed.
4. What kind of transplant did he/she receive? How knowledgeable were you of what that meant?
5. From a caregiver's perspective did you feel you were properly educated about what your role would be after transplant, especially in the first 100 days?

Daily Routine

1. Walk me through a normal day for you and your <u>[insert relationship of patient]</u>.
2. I'd like to start by asking you about [patient's] routine. What are the biggest changes to <u>[insert relationship of patient]</u> daily routine since diagnosis?

Outpatient Care Activities – ask them about how they came up with their current system

1. Do you have any required self-care at home? Such as central line, oxygen machine, physical therapy, etc.
    a. How was the discharge education? Was that helpful to prepare you for what would happen next?
    b. Tell me about any challenges you have had performing these tasks at home?
    c. Have you had any challenges recalling specific instructions from clinicians when you perform these tasks at home?
2. How many medications is he/she on?
    a. How does he/she store medications?
    b. How does he/she remember to take medications?
    c. Do you have any reminders for him/ her?
    d. How do you know when he/ she needs a refill on medications?
    e. Do you wish you had more assistance from the hospital during the transition from inpatient to at home care in regards to the medications?
3. Do you track/monitor his/her diet, fluid, or other specific things to inform clinicians?
    a. How do you keep track of them?
    b. Have you had to modify your own diet in response to patient needs and restrictions?

Resources and Organization

1. When you have a medical question or concern at home, what do you normally do?
    a. Are there any useful resources for you? (i.e. peer support group, online forum, family or friends)
2. Tell me about how you organize your medical record or important health information at home?
    a. How do you store and organize them?
    b. What information do you usually refer to in those documents?
3. What digital devices do you use on a daily basis?
    a. Do you use them to find or exchange medical information with your [insert relationship of patient]?
    b. Do you use social media to share medical updates with others?
    c. What is the reason for using/ not using particular device or social media?

Local/ At Home

1. If you are required to stay locally (w/in 90 mile) did the hospital provide you with information on restaurants and activities that you can do?

2. Can you tell me about your transition from home to a local location?

  a. What are things that would be helpful to know about in this new location i.e. gyms, restaurants, caregiving groups, etc.

Now we're going to transition to talking more about the expectations caregivers face in general…

Caregiver Burden

1. Tell me about some of the big changes to your daily routine you've experienced since diagnosis.
2. How has his/her illness affected you and your family? (i.e., relationship with your friends and other family members)
3. Could you describe your relationship to [insert relationship of patient]?
    a. What kind of expectations do you have from each other?
4. Do you engage in any activities to relieve any stress you might have?
    a. If yes, could you describe what you do?
    b. And how often would you say you take part in those activities?
5. If you have a day-off from caregiving, what do you usually do?
    a. If not, what would you want to do during a day off?
6. Tell me about any available peer-support groups or mental wellness programs for caregivers you may have heard about that are provided by hospital? If a local support group existed would you be likely to attend? Would you be interested in knowing who other patients/caregivers are on the floor as a form of support during and after the process?

What advice would you give new caregivers before they begin the transplant process?

. . . .

We are exploring the possibility of developing a program for caregivers of BMT patients. The goal would be to enhance the well-being of caregivers as they navigate this challenging time.

One of the ideas we are exploring is to expand an existing educational or informational app to allow you to journal your experience throughout the first 30 days while your patient partner is in the hospital. How would you feel about that? (Do you think that this is a good idea? If so, why.)

Other activities we are considering are those that have been shown to increase well-being and resilience. I would like to share with you 6 such activities and see which ones you think might be good for caregivers to try once your patient partner has been discharged from the hospital. Please rate each one on a scale of 1 to 10 with **1 indicating that you would not likely engage in this activity and 10 being very likely to engage in this activity.**

Savoring: In this exercise, you would be asked to spend a few minutes each day savoring at least two everyday experiences (e.g., morning coffee, the warmth of sunshine, a call from a friend). You are to be mindful (very aware of the moment), while savoring the experience and use all of your senses (sight, hearing, smell, taste and touch) to solidify the memory.

Please rate this savoring activity on a scale of 1 to 10 with 1 indicating that the activity would likely be a bad idea to 10 indicating that the activity sounds like it could be very beneficial.

Positive Piggy Bank: In this activity, every evening you will think about the things that made you happy that day. You will write down one of these moments on a piece of paper, fold up this piece of paper and drop it in a piggy bank. (We will provide the piggy bank.) At the end of 30 days, you will "close your account," which means that you will open the piggy bank and read and savor all the deposited happy memories.

Please rate the Positive Piggy Bank activity on a scale of 1 to 10 with 1 indicating that the activity would likely be a bad idea to 10 indicating that the activity sounds like it could be very beneficial.

Random Acts of Kindness: Although we typically do many kind things every day, we often do not set out to intentionally do kind things for others or even ourselves. One day this week, do 5 acts of kindness – be sure to do something kind for yourself. Self-care is important, too. Perhaps, you could take a long bubble bath, go for a walk in the park, enjoy a Popsicle, or sleep an extra 20 minutes.

Please rate Act of Kindness on a scale of 1 to 10 with 1 indicating that the activity would likely be a bad idea to 10 indicating that the activity sounds like it could be very beneficial.

Using Your Signature Strengths: Your top seven character strengths would be identified using a brief questionnaire. These strengths could include things like kindness, creativity, perseverance, bravery, intelligence, and many others. You will then be directed to use one of these strengths in a new way every day over a week.

Please rate this strengths-based activity on a scale of 1 to 10 with 1 indicating that the activity would likely be a bad idea to 10 indicating that the activity sounds like it could be very beneficial.

Pleasant Activity Scheduling: For this activity, you would set aside a small block of time each day for a positive activity. This could be as simple as watching a favorite show, taking a bubble bath or having ice cream with a friend. You are to treat this "appointment" with same seriousness as you would other appointments on your calendar.

Please rate Positive Activity Scheduling on a scale of 1 to 10 with 1 indicating that the activity would likely be a bad idea to 10 indicating that the activity sounds like it could be very beneficial.

<u>Gratitude Journal</u>: Every day for 30 days you will write down three things for which you are grateful. The challenge for you is that every day you will write down three new things. Be sure to keep your eyes open for life's simple pleasures.

Please rate keeping a Gratitude journal on a scale of 1 to 10 with 1 indicating that the activity would likely be a bad idea to 10 indicating that the activity sounds like it could be very beneficial.

<u>New Questions:</u>

1. As a caregiver if we were to create this app what sorts of things would be most beneficial for you?
2. As a caregiver what do you need most on a day to day basis?
