# Supplementary material for: A Mobile Health App (Roadmap 2.0) for Patients Undergoing Hematopoietic Stem Cell Transplant: Qualitative Study on Family Caregivers' Perspectives and Design Considerations
Source: JMIR Mhealth Uhealth. 2019 Oct 24;7(10):e15775. doi: 10.2196/15775 (PMC6913725; doi:10.2196/15775)
Supplement: Multimedia Appendix 6 [file mhealth_v7i10e15775_app6.pdf]

Multimedia Appendix 6

Code Book

Code	Abbreviation	Definition	Example
Information Source	SOURCE	A book, website, person, etc., containing facts or guidance about health and/or the process of BMT.	Interviewer: "Where have you been getting your information about her condition and the bone marrow transplant?" Male Respondent: "The internet cancer websites."
Information Sharing	SHARE	An interviewee references the act of disseminating facts or guidance about health and/or BMT	What he's been doing is, he's making a copy of his counts and sending those to me through the phone. I can at least see the (02:28 inaudible) of how he is recovering, if his counts are going down, and if any are recovering.
Listening	LIST	An interviewee discusses auditorily absorbing information about health and/or BMT.	"They always explain stuff very well. I had one nurse do my chemo today zero to transplant, what I'd be feeling like. She didn't even have to look it up, she knew it, right in her head. She walked over and drew the dip, and she goes, "See, you're going to be right here. Then after this then you start going this way." She explained it good."
Reading	READ	An interviewee discusses absorbing information about health and/or BMT from a textual source.	"They gave us the book, and then reading up on it scared the daylights out of us."
Map Use	MU	Any reference to time spent interacting with the Roadmap application.	"I just looked at it today for the first time..."
Social Media	SOC	Any reference to time spent interacting with Facebook, Instagram, etc. in a manner related to health and/or BMT	" I recorded the transplant and sent it to them. I posted it on Facebook, and so everybody got to feel like they were apart of it", "We actually have a group thing on Messenger. Anytime we have an update, everyone knows right away."
Medication/ Treatment	MED	Specific to BMT	"They put me on Cytoxan and high doses of prednisone."

Symptoms	SYMP	Symptoms and Side Effects Specific to BMT	" I got mucositis. Everything was cruising until that point."; "He has GVHD of the skin, so he gets lotion three to four times a day. His blood pressure is high. He's on steroids, so I take his blood pressure three times a day."
Labs	LAB	Specific to BMT (include imaging)	"[...] it's cool too you get to see your counts before the doctors actually tell you."
Medical History	MH	Specific to Cancer Diagnosis	Yes, it's AML. That's Acute Myeloid Leukemia. It's a difficult cancer, and it's difficult to treat. That's why he's having the transplant.
Previous interaction with healthcare system	PINTER	Non-specific. Reference to any healthcare encounter prior to the current treatment course/transplant	"I actually had an experience where I knew what I was supposed to get and wasn't getting it and called them on it."
Planning ahead	PLAN	An interviewee discusses a specific self-directed strategy for pre- and/or post-BMT care or coping (including mental wellness); Do not double code with D2D	I write down information. Every day that I come into the hospital I start a new day, and I put the date and the day, who I talk to, who came in, what happened, what questions were asked, the answers, what medication changes, anything that's happening that day.

Caregiver Duties/ Responsibilities w/o burden	CARE	Any interviewee mentions and/or makes evaluative statements about their obligations in the caregiving process (i.e. they say how they feel about aspects of the caregiving process or caregiving responsibilities)	"I usually try to keep track of his counts, his meds", "He’s on steroids, so I take his blood pressure three times a day. He takes 13 pills in the morning, 12 at night, two in the afternoon.", "We make sure to keep, on hand, the blood pressure monitor, the thermometer.", "She’s had ports, she’s had two kinds, she’s had (03:49 unclear). I can do all of that. I would never have known a long time ago that I could do it, but I do. I administer all that to her.", "Trying to get him a little active, and bathing.", "Our goal is four bottles of water a day. I make sure he drinks those.", "I want to take the best care I can of her. And obviously, I don’t want anything to happen to her. I do everything I can. I try to do the best I know how to make sure she’s healthy. I get her to her appointments and make sure I set up her medications at home, her daily... She has seven days a week, AM/PM box and I set those up for her every week. I make sure she’s getting the right medication, the right doses."
Caregiver Duties/ Responsibilities w/ burden	CARE w/B	Any interviewee mentions and/or makes evaluative statements about their obligations in the caregiving process AND suggests that these obligations constitute a strain/hardship.	"Luckily, the nurse will come out and show us again. I didn't go to school to be a nurse, holy smokes"
"Living day to day"	D2D	An interviewee discusses being present/ focusing on the situation at hand as a method of coping with the BMT process (do not double-code with PLAN)	"Interviewer: Do you feel kind of, coming in, this day, that you have a good understanding of what to expect during this month in the hospital? Interviewee: No, I still just go day by day. Interviewer: And do you think that’s best for you? You think, knowing about the whole thing at once would be a little too overwhelming? Interviewee: Yes. Just want to take it one day at a time."

Technological issues	TECH	An interviewee discusses using/ not using technology to share/ find/ learn medical information	"I think I went on the computer when we first got diagnosed with it.", "Be The Match website, because I watched a lot of videos and I got a lot of information about the transplant. More than the book.", "His doctor's appointments are set to my cell phone, which is really convenient, so I usually just use the same thing for his appointments.", "We get the weekly printouts from here. All of her medication, I keep a list in my phone, so I know exactly what she's taking, how many milligrams, how often. And every time they change it, I update it in my phone. That way I always have it with me no matter where we are.", "I would say is on our phone or on the iPad checking things out with the portal."
Chemo brain	CB	An interviewee mentions poor cognitive functioning or cognitive deficits due to chemotherapy.	It's not that I don't feel he's being truthful, but there are some periods where his memory has lapsed, because he's in a chemo fog. He gets easily overwhelmed with a lot of information.
Knowledge as Power	KAP	An interviewee suggests that being well-informed about BMT makes them more comfortable navigating their care process.	"I've done a lot of research and I continue to do a lot of research through a variety of online sites, anything I can get my hands on to read about it, I read about this. And different forums and see what different people are coming up with. The more that I read, the more comfortable I become."
Knowledge as Burden	KAB	An interviewee suggests that being well-informed about BMT makes them less comfortable navigating their care process.	I don't like too much information is bad. You get too much information you forget how to use your common sense. It's that simple.
Transportation/ far distance from the clinic	DIS	An interviewee discusses transportation or distances from home to the clinic	"We don't live in town, we live out of town, but we have an East Lansing address. We live out of the city.", "Yes. Driving here, the traffic depending on the time of the appointments can be challenging with the winter weather."

Changes in daily/ home/ family life after diagnosis	CHAN	An interviewee discusses changes in her daily life after diagnosis or transplantation	"Not only our home, because it was set up like a hospital room when she was home", "She has a little brother. He was separated from her. I never got to see him either.", "We came back home. I felt like it was a stressful environment, because there was drywall everywhere. It didn't feel homey. It felt like, "There's a project at home that we've got to take care of, but we don't have time. I have to focus on her now.", "I've got closer with my significant other."
Communication with care team	COMM	Any reference to communication (any aspects of communication itself and tool) with care team	"There's an afterhours number that you can call and usually, a doctor will call you back within 30 minutes.", "One of his doctors has given me her number so if anything is urgent she doesn't mind.", "If we have questions at home, I'll call in."
Medical information organize system	ORGSYS	An interviewee references the act of organizing/ tracking health or medical information	"We have a filing cabinet. Each member of the family has their own section and any of the medical stuff will be in a folder.", "I have this folder that I throw after visit summaries in. I keep a few notes."
Medical questions	QUES	An interviewee discusses any health/ medical questions that they may have	"If anything has changed sugar-wise, any new rashes or anything, I try to let them know or ask questions.", "Actually, I have a notebook and I have questions in there about anything that's come up or medication changing or things like when things will change. Questions like that. I pretty much get them answered and it's helpful.", "Then obviously more questions came up because they added different medications.", "We have a visiting nurse that comes once a week. I've been asking her questions."
Personal info (background information)	BACK	An interviewee mentions her background	"I'm a stay-at-home mom, so it's my job", "I am divorced and I have a boyfriend who has been in her life for four years.", "I am a software developer. I went to school here at U of M."
Issues during the clinic visit	CLINIC	An interviewee discusses any issues related to outpatient clinic visit	"Time management is hard. You wait an hour, hour and a half, two hours. And then you wait to be seen. If you're pressed for time, it's hard."

Religious/ belief	REL	An interviewee discusses her religious or belief as a way to stay strong, navigate tough journey, etc.	"We pray every day. We don't only pray for yourself, we pray for everybody. We have a lot of friends that need prayers, so we pray for everybody", "You know, and I think the Lord prepared me for the time as this."
Stress management strategy/ Coping strategy	SM/COPE	An interviewee discusses her own strategy as a way to navigate tough journey, etc., and stress management activities	"I'd tell them to keep their sense of humor and a positive attitude. It sounds cliché but take it one day at a time.", "listen to everything the doctor and the clinic people in the clinic tell you to do. Read all the information they give you so you will be prepared and do what they say to do, because I am certain there are people who are not drinking distilled water and are not the level of clean that we've got.", "You have to be there and you have to understand, this is something that you have to give 100 percent, and you do it out of caring anyway.", "I just took it as it came and we worked through it together. She's the champ."
Peer support/ Mental wellness group	PEER	An interviewee discusses involvement/ participation in peer support group offered by hospital or foundation	"When we were in-patient, they had meditation on Tuesdays and Wednesdays. The family gets together in the lounge, and they have coffee and talk.", "I'm looking for a support system for people with the same disease, because it's one thing for us."
Family and Friends support	FFS	An interviewee discusses support from family and friends over the course of illness	"It's just gone well. He fit into the role real well as did my sister, my girlfriend and my sister-in-law. Everybody has done super. I hope everyone would have the support I have had."
Satisfaction with care or care team	SAT	An interviewee discusses positive aspects or benefits of her care team	"I can't remember her name, woman answers the phone every time. (Laughs). She's very nice. You get called back almost right away.", "Everybody [in clinic] is really nice"
Positive emotion/ feelings	POS EMO	Any interviewee mentions or makes positive statements about their feelings in the caregiving process	"I feel pretty comfortable doing everything [with IV].", "I think we've both been pleasantly surprised that he's been able to do what he is on his own."

Negative emotion/ feelings	NEG EMO	Any interviewee mentions or makes negative statements about their feelings in the caregiving process	"We've been given permission to travel, but the other kids are in school, stuff and we're still nervous.", "But it's very overwhelming-", "That's been very stressful from that standpoint.", "I'm not comfortable doing it"
Financial burden	FIN	Any interviewee mentions financial issue and indicates that these issues constitute a strain/hardship	"Financially, it's been awful."
Caregiving benefit	CARE BEN	Any interviewee mentions and/or makes positive evaluative statements about their obligations in the caregiving process AND suggests that these obligations constitute a benefit/ positive aspect; Do not double code with POS EMO	"He is much easier to help (laughs) than my dad was.", "I'm getting stronger", "It's just gone well-Everybody has done super. I hope everyone would have the support I have had."
Caregiving burden w/o financial burden	CARE w/B (neg)	Any interviewee mentions and/or makes negative evaluative statements about their obligations in the caregiving process AND suggests that these obligations constitute a strain/hardship; Do not double code with FIN or NEG EMO	"just changing the dressing has been a source of angst for me. That's really difficult.", "The other thing is you just don't realize when they want 24/7 care, it's around the clock."
Caregiver's desire	DES	Any desire that an interviewee has, but hard to do under current situation	"Actually sleeping, not worrying. Having my stress escape. All day,", "I'd probably like to go out of town shopping for the day, I guess. That's probably what I'd like to do.", "Take my kids out, because I don't have that time to do that."
