# Supplementary material for: A Mobile Health App (Roadmap 2.0) for Patients Undergoing Hematopoietic Stem Cell Transplant: Qualitative Study on Family Caregivers' Perspectives and Design Considerations
Source: JMIR Mhealth Uhealth. 2019 Oct 24;7(10):e15775. doi: 10.2196/15775 (PMC6913725; doi:10.2196/15775)
Supplement: Multimedia Appendix 7 [file mhealth_v7i10e15775_app7.pdf]

Multimedia Appendix 7

A – F. Artifacts Collected in the Home environment

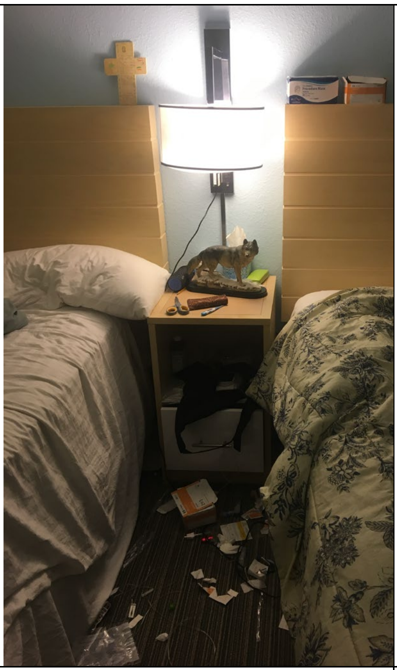

A. Small living conditions of temporary housing (e.g., hotel room); CG09.

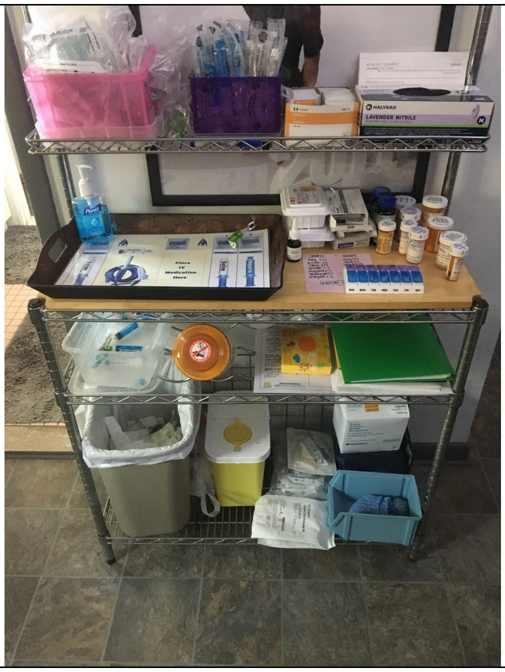

B. Medication organization; CG02.

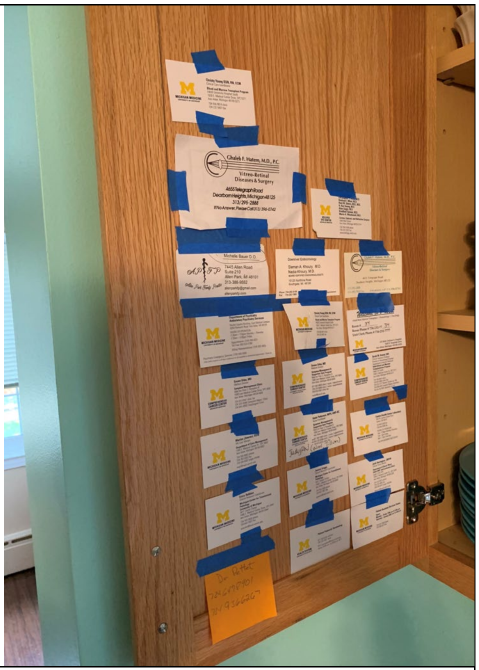

C. Business name cards posted on the kitchen cupboard; CG24.

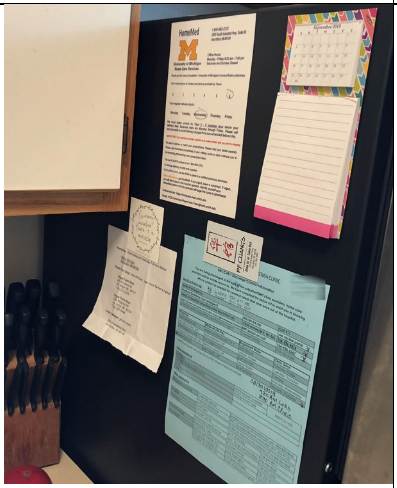

D. List of local area restaurants and grocery markets, and discharge instructions posted on the refrigerator; CG13.

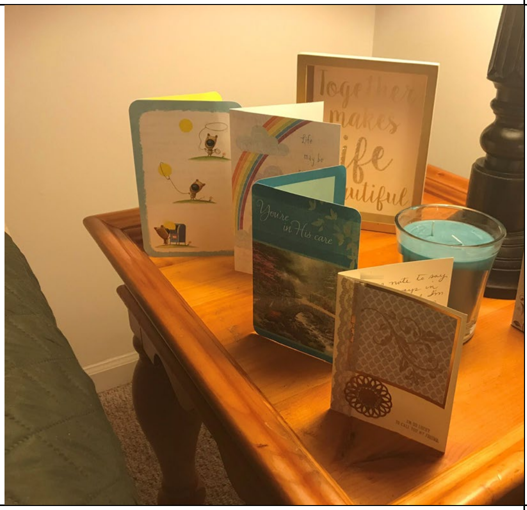

E. Get Well cards provided encouragement and support; CG05.

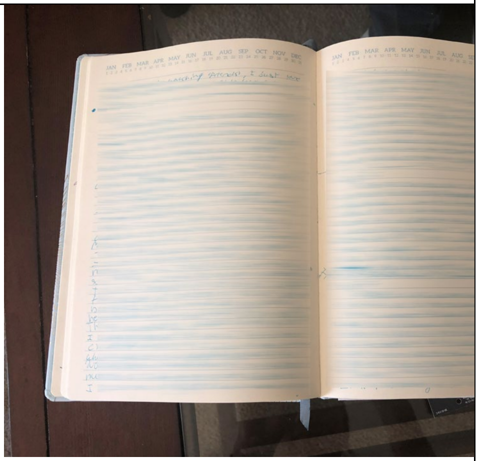

F. Journaling provided an opportunity to chronicle the transplant journey; CG01. *The text has been blurred to preserve privacy and confidentiality.*
