# Supplementary material for: A Mobile Health App (Roadmap 2.0) for Patients Undergoing Hematopoietic Stem Cell Transplant: Qualitative Study on Family Caregivers' Perspectives and Design Considerations
Source: JMIR Mhealth Uhealth. 2019 Oct 24;7(10):e15775. doi: 10.2196/15775 (PMC6913725; doi:10.2196/15775)
Supplement: Multimedia Appendix 8 [file mhealth_v7i10e15775_app8.pdf]

Multimedia Appendix 7

Participant quotes

A. Stress related to balancing caregiving duties

“The home life has the largest impact because our lives get disrupted. Fortunately, my son is a junior in high school, he’s mature and independent. (Patient’s name) is more of an adult, so I don’t know how it would be… I’ve seen people in the hospital who have these little children and the children have a hard enough time understanding what’s happening to them, let alone the parents. So, in our case, I would say that the largest challenge, the biggest challenge has been the home life, learning to balance just everything, bills, priorities. My son, one thing we had to do before coming here is my son has a job, how is he going to get to his job? I was the one driving him back and forth. We fortunately have a friend of ours sold a car to us really cheap and my son is thrilled. (Laughter.) I’ve got wheels.” [CG01]

“A normal day starts with going over in the morning making sure he took his meds, making sure he does his blood sugar. Did all of the fun little things that he’s supposed to do. Hook him up to the port, doing the flesh on the two ports. And then hooking up the machine on the third port, and then getting him set. Making sure he’s got what he needs, that kind of thing. Throughout the day we checked on him, make sure he’s done whatever needs to be done, pillows, blood sugar. Then after that in the evening when we take off the machine we prep to make sure we have enough product for the next day. So we pull out all the syringes that we need, the heparin and the saline. And then make sure we have the bag ready for the next day and that fun stuff. And then go over in the evening and make sure he took his nighttime pills.” [CG14]

“Unless he's having cereal, get him breakfast. Then I eat. Then we go for a walk and then I set out his magnesium. Then do my shower and get him ready for his shower. Then it's usually time for 9:00 meds and then we do his eye drops. Then we try to go for a walk again, unless it's too hot out. He has to have a little snack. Just try to repeat the routine. Cleaning, which thank God I like to do because I keep the place clean. Right now, sheets are washing. Making sure everything is clean and sterile for him. If he's doing well enough, if he doesn't feel like it, I leave him here and go to the Meijer, which is real close or Rite Aid to get prescriptions or to get stuff like that done.” [CG15]

“For the most parts of days are still pretty normal for the most part, except I'm working weekends now. I only work three days a week. I'm only working two now with using the FMLA. But I pretty much get up and go about my day as normal. It only takes a couple of minutes really to set up his IV stuff and then every week, after he gets his Tacro level dosed, I just go through do his weekly pills and put him in a little planner thing. That only takes 10 minutes on Thursdays also. The biggest thing would probably be going out and doing things by myself. I was a vegetarian when we met, so I go to the grocery store now and I'm not entirely sure how to purchase meat and compare it. That's always been his thing.” [CG19]

B. Learning and adapting to new routines (resiliency)

“There’s a help number. And, just repeating it over and over, now I can do it in my sleep practically. But those first couple of weeks, it was scary.… Oh, changing her dressing, the first time I had to do that was frightening too, because now you’re messing with the line here and you have to be totally sterile. It freaks (patient’s name) out because I have to pull and she’s like, ‘You’re going to pull my line out.’ (Laughter.) I think just talking through as we’re doing things…” [CG01]

“[The challenge was] really just getting a routine, getting set, putting this on, taking it off, flushing it, and that's the biggest thing, because you got one of those stuck down in here. It's not normal…It's been a challenge for me because I have it all written down, what to do, and it took me weeks to not have to have that paper. Thankfully his memory is better than mine, so he'd stopped me. ‘No, we have to do this first.’ That was getting used to doing that and make sure I'm sanitary and all that, because it can obviously set him back if we're not.” [CG21]

“I don’t know how to put that into words. I guess we don’t think that it’s not going to work, so it’s just like a bump in the road to us. It’s not like it was the end of the world. It’s just a tough time that we have to get through. We’re working together…. It’s just temporary. We’re going to get back to where we were. We used to travel a lot and do a lot of things. It’s just a bump in the road. I think it’s made us think, too.” [CG05]

C. Balancing one’s own needs with the patient’s needs (insight)

“Sleep. I try to golf. I try to have my time that’s difficult, but I have to schedule it in around the med stuff, so there’s a window when I can do stuff and so I have to do it in that window… I’m really getting to [golf], once every other week. I used to play minimum weekly so that’s a bit of a sacrifice.” [CG08]

“This is such a traumatic life event that happens, cancer just completely takes over your life and in order to maintain your sanity you got to have and do something and get out of the house and try to feel like a normal person.” [CG08]

“I use friends a lot because they know me. I find them to be good therapists for these sorts of things.” [CG22]

“Yoga, I do yoga. I drink wine. I read, I play video games, I talk to friends, I talk to my mom. I like to take walks, I ride bikes. I do a lot of things to try to relieve stress.” [CG22]

D. Benefits of caregiving

“Yes, you’re away from home more.… I think it’s just brought us all closer together. I think it’s made them even realize more of we’re here, we’re not going anywhere. We're the ones you can count on type thing.… You realize what’s important and what’s not.… But you realize that too, that it’ll never be totally the same. Things will always be different while she's recuperating.” [CG13]

“It’s just a tough time that we have to get through. We’re working together. His daughter, he was married before and had two daughters. We don’t have any. His daughters, I think that’s brought us closer, and his mother. We were always close, but now but now we talk every day, an hour. Before I wouldn’t call her and she wouldn’t call me. It’s brought me and his family close together. Being down here, I miss my family more. My mom is 85 so I’d usually go over there every two or three days to check on her. Now I can’t, so I’m having my two brothers, I told them to keep an eye on her, go over there, keep close to her…I’ve even said to other people, if somebody’s having a bad day, you see somebody and they’re crabby, don’t be mean back to them because you don’t know what they’re going through.” [CG05]

E. Positive activity interventions

“I have to do [savoring the moment]. That is why I get up at five every day and I don’t get him up till eight. That’s my morning time. Watch the news, coffee, send my wife off to work, feed these guys, play with them, that’s my time then. You have to have it. If you don’t have it then you’ll lose” [CG24]

“Yes. I read books, so just having personal reading time. The suggestion about having two activities, yes. For a while, I had brought myself a Nintendo and that helped me a lot, just keeping things in order. I’d put that at a 10 too because the journaling could be one of those things.” [CG10]

“[Using signature strengths would be] very nice, because then you get to be aware of your own strengths, weaknesses, and stuff like that…I’d give that a 10…Yes, especially as a patient. You feel like you’ve lost your identity. If you know what your core is, it helps you rebuild yourself a little bit better.” [CG10]

“I’ll rate [Positive Piggy Bank] a six. I don’t think I would find that helpful, but I can see a lot of people that would. Just sometimes, someone has a bad day just the thought of something good might help them fall asleep in a better mood.” [CG02]
